# A randomized controlled trial of a multi-component family intervention to lower depression and address intimate partner violence (MILAP) among young married women in Nepal: a study protocol

**DOI:** 10.21203/rs.3.rs-7048952/v1

**Published:** 2025-09-09

**Authors:** Mina Shrestha, Elsa Heylen, Kripa Sigdel, Meghnath Dhimal, Prajwol Nepal, Prakash Pant, Rajesh Kumar Jha, Rajiv Rimal, Rekha Khatri, Saugat Joshi, Shuvam Sharma, Srijana Shrestha, Sumitra Poudel, Sunita Mainali, Sabitri Sapkota, Bibhav Acharya

**Affiliations:** Possible; University of California San Francisco; Possible; Nepal Health Research Council; UNC Gillings School of Global Public Health: The University of North Carolina at Chapel Hill Gillings School of Global Public Health; Possible; Possible; Johns Hopkins University; The University of Sydney; Possible; Possible; Wheaton College (MA): Wheaton College Massachusetts; Women’s Rehabilitation Center; Women’s Rehabilitation Center; Possible; University of California San Francisco

**Keywords:** Intimate Partner Violence, Depression, MILAP, Family intervention, Mother-in-Law, Randomized controlled trial

## Abstract

**Background:**

Intimate Partner Violence (IPV) is a well-established driver of mental health problems, often doubling the prevalence of depression. In Nepal, approximately one in four women experience IPV, with young women disproportionately affected by mental health issues. Many women in Nepal reside with their mothers-in-law (MILs), who can influence violence and restrict women’s mobility, highlighting the importance of including them in interventions targeting IPV. To address this, we developed a Multi-component family Intervention to Lower depression and Address intimate Partner violence (MILAP) and will conduct a randomized controlled trial (RCT) to evaluate its effectiveness in reducing IPV and depression.

**Methods:**

This RCT will enroll 300 family triads, each comprising of a young married woman (aged 15–24), her husband, and her MIL. Blinded staff will conduct baseline assessments, after which each triad will be randomly assigned to either the MILAP or Enhanced Usual Care (EUC). MILAP comprises nine sessions delivered by trained psychosocial counselors and focuses on strengthening the supportive relationship between MILs and daughters-in-law (DILs), providing behavioral couples therapy, and enhancing overall family dynamics. EUC includes standard care, such as individual and group counseling for IPV, enhanced with crisis counseling and referral support to ensure participant safety and access to additional resources. All participants, regardless of intervention allocation, will be assessed at baseline and at 1, 3, 6, 9, and 12-month follow-ups. These assessments will measure: primary outcomes [depression using Patient Health Questionnaire-9; IPV using Indian Family Violence and Control Scale], secondary outcome [post-traumatic stress disorder (PTSD) using PTSD CheckList-Civilian Version], and other outcomes. We will also conduct serial in-depth interviews to explore the mechanisms underlying MILAP’s effectiveness and perform a cost-effectiveness analysis to assess its potential for scalable implementation.

**Discussion:**

MILAP is a culturally adapted, family-based intervention designed to reduce IPV and depression among young Nepali women by improving communication, strengthening coping skills, and fostering supportive family relationships. This trial will assess MILAP’s effectiveness, ethical rigor, cultural relevance, and scalability, with the goal of reducing IPV and improving family dynamics in low- and middle-income settings.

**Trial registration:**

This trial is registered in ClinicalTrials.gov with the National Clinical Trial (NCT) number: NCT06834867; first registered on February 24, 2025.

## Introduction

### Background and rationale {6a}

Intimate partner violence (IPV) and mental health problems pose major global public health challenges, particularly in low- and middle-income countries (LMICs), where one in three women experiences IPV.(1–4) IPV is defined as abusive control or psychological, physical, and sexual violence perpetrated by an intimate partner.(3) It is a well-established contributor to mental health problems, often doubling the prevalence of depression, increasing social isolation, and escalating the risk of post-traumatic stress disorder (PTSD).(5–12) In South Asia, 25–50% of women who experience IPV report depressive symptoms.(13–16) In Nepal, our study site, IPV affects 26% of women.(17) Despite ongoing IPV, young women in LMICs are often unable or unwilling to pursue divorce or separation.(18, 19) Young women in Nepal face heightened vulnerability, with half marrying before the age of 18 (20) and experiencing disproportionately high rates of mental health issues. Among women of reproductive age, suicide is the leading cause of death. (20–24)

There is evidence that persistent IPV perpetuates mental health problems, severely hinders recovery, and heightens suicide risk.(7, 25–27) Existing interventions addressing IPV and mental health issues in LMICs are scarce and often ineffective.(28–31) Currently available interventions, such as cognitive behavioral therapy, have shown effectiveness primarily among women who have already exited relationships that led to IPV (32–34) but may be less effective for those who remain in such relationships and continue to experience violence. Therefore, there is an urgent need for culturally appropriate, evidence-based interventions that simultaneously reduce IPV and improve mental health.

Family-based interventions offer a promising approach in LMICs, where IPV and mental health issues often extend beyond spousal relationships, as many married women in LMICs reside in households with in-laws, and mothers-in-law (MILs) often influence IPV and restrict the freedom of movement (FOM) of daughters-in-law (DILs).(1, 35, 36) Our prior study found that positive MIL-DIL relationships can enhance spousal and family dynamics, and improve women’s (DILs’) mental health. (37) Despite this, most interventions focus solely on women (38–42), occasionally involve husbands (16, 43–49), but often overlook the broader family context. Such interventions may unintentionally place the burden of change on women, rather than creating a supportive home environment where they can access support from their MILs. Furthermore, if women access IPV services independently, it may escalate IPV if perpetrators become aware. In addition, many women might hesitate or avoid participating in IPV interventions due to the stigma associated with disclosing IPV, fear that disclosure may worsen the violence, and a desire to maintain and protect the family unit and honor. (50–53) Moreover, men who engage in IPV often normalize their behavior and may be less receptive of any intervention explicitly presented as targeting IPV. Given these sociocultural circumstances, it is essential to incorporate MILs into intervention strategies and focus on a non-stigmatizing approach that promotes communication and relationship-strengthening skills within the family.(16, 44, 46–49)

To address these gaps and the need for culturally appropriate family-based interventions, we developed the **Multi-component family Intervention to Lower depression and Address intimate Partner violence (MILAP) among young married women aged 15–24 years**. MILAP **(“MEE-lahp”) means** “unity and reconciliation” in Nepali, and is a **nine-week family-based intervention**involving the young married woman, her husband and her MIL. It consists of **four combined MIL-DIL sessions for the woman and her MIL focusing on gender norms, allyship and FOM, four sessions of behavioral couples therapy (BCT)**for the woman and her husband, and **one triad summary session** involving all three family members (woman, husband, and MIL).

**Figure 1:**
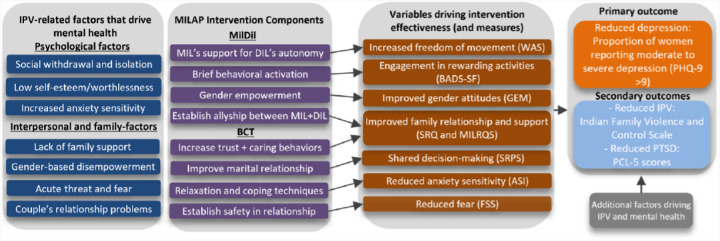
Primary hypothesized pathways for MILAP to address IPV-related factors driving mental health outcomes

MILAP is informed by Social Cognitive Theory (SCT) (54–57) and findings from our pilot studies (Figure 1). SCT offers a culturally appropriate model that emphasizes interpersonal dynamics, the role of family environments in shaping behavior, and strategies to reduce barriers while enhancing facilitators of healthy behavior change.(55, 58) As women in the study setting have fewer options to leave their marriage (19), are heavily affected by the views and behaviors of their family members (37), and are committed to staying married, SCT allows us to incorporate these factors at the women’s individual, interpersonal and family levels. Given that IPV contributes to depression through mechanisms such as social isolation, restricted mobility, diminished self-efficacy and negative family dynamics, MILAP specifically targets determinants identified as modifiable in our prior research.(6–12, 59). Evidence supports the integration of IPV and mental health interventions that build problem-solving skills (60), strengthen relationships with key family members (61, 62), promote social support (63), and enhance resilience and empowerment.(64) MILAP addresses IPV and depression in a non-stigmatizing and depersonalized manner, and offers coping skills for women, mutual empowerment of MILs and daughters-in-law (DILs), and enhanced spousal communication and trust.

We conducted two single-arm pilot studies to test the acceptability, feasibility, and preliminary impact of MILAP, and found it to be well-accepted and effective in addressing IPV, depression and PTSD among young married women.(65, 66) In this manuscript, we report the protocol for a community-based fully-powered two-arm randomized controlled trial involving 300 families to evaluate the effectiveness of MILAP in reducing IPV, and improving depression and PTSD symptoms among **young married women aged 15–24**. Moreover, this randomized controlled trial will include cost-effectiveness analyses to evaluate the health and economic benefits of investing in MILAP, thereby assisting policymakers and payors in assessing MILAP’s potential for future scalability.

### Objectives {7}

The primary objective of this study is to evaluate the effectiveness of our novel family-based intervention MILAP on IPV, depression and PTSD among young married women aged 15–24 compared to those receiving enhanced usual care (EUC). To acheive this objective, we have defined three specific aims:

1. Conduct a 12-month randomized controlled trial to assess the effectiveness of MILAP on IPV, depression and PTSD among young married women aged 15–24 in Nepal.
2. Conduct a mixed-methods assessment of mechanisms of change for MILAP’s effectiveness.
3. Conduct a cost-effectiveness analysis of MILAP for IPV and depression.

### Trial design {8}

This is a community-based, randomized controlled trial in which family triads (comprising the woman, husband, and MIL) will be randomized 1:1 to either the intervention (MILAP; n=150 triads) or the comparison (EUC; n=150 triads) group.

## Methods: Participants, interventions and outcomes

### Study setting {9}

We are conducting this study in three districts of Nepal: Sunsari and Udayapur districts of Koshi province, and Sarlahi district of Madhesh province. We selected these sites because of their relatively higher prevalence of IPV (17, 67), and the existing networks and footprint of Women’s Rehabilitation Center (WOREC), which is a national non-profit organization focused on violence against women and children. This study will leverage WOREC’s networks for participant recruitment. We have obtained approvals from the Institutional Review Board of the University of California San Francisco [23–40311] and the Ethical Review Board of the Nepal Health Research Council [120–2024] to conduct this trial in these study sites.

### Eligibility criteria {10}

We will assess the eligibility of participant using the following inclusion and exclusion criteria.

#### Inclusion criteria

1. Married woman aged 15–24 years, living with her husband and MIL.
2. Living in the catchment area with no stated intention of leaving during the study period.
3. Participant speaking Maithili or Nepali language, as these two are the most common languages spoken in the study sites.
4. Wife reporting IPV (physical, sexual or abusive control) in the last 12 months assessed by three questions from the International Violence Against Women Survey (IVAWS). (68)
5. Wife expressing desire to remain in the current relationship/family.

#### Exclusion criteria

1. Any participant with severe alcohol dependence defined by the Severity of Alcohol Dependence Questionnaire (SADQ) score ≥ 31.(69) Any participant with mild to moderate dependence will be referred but not excluded.
2. History of IPV severe enough to result in hospitalization in the past 12 months, as MILAP is not designed to address severe levels of violence. Any participant reporting severe IPV cases requiring hospitalization will be referred to tertiary care facilities.
3. Any participant with severe depression, defined by the Patient Health Questionnaire-9 (PHQ-9) score>19 and/or high suicide risk assessed by ninth question of the PHQ-9 (“thoughts that you would be better off dead or thoughts of hurting yourself in some way”) (70) and the Nepal Emergency Suicide Screener-3 (NESS-3) adapted from the Ask Suicide-Screening Questions(71), will be referred to specialized mental health centers for assessment. After discharge from the healthcare facility, they will be approached again for eligibility.
4. Woman who is pregnant; since pregnancy changes the family dynamics and in our study settings, pregnant woman often temporarily moves back to live with their parents where the source of IPV is no longer around. However, if any participant becomes pregnant during the study, she will continue in the study.
5. Cognitive problems/disability precluding participation as self-reported by participant.

### Who will take informed consent? {26a}

Research assistants, trained in research ethics, will screen young women based on the inclusion and exclusion criteria outlined above. Once a woman is confirmed to be eligible and provides informed consent, the research assistant will proceed to screen her husband and MIL. The research assistant will then obtain written informed consent individually from the woman, her husband and MIL.

### Additional consent provisions for collection and use of participant data and biological specimens {26b}

In addition to providing consent to participate in the study, the consent form includes an option for participants to opt in to participate in serial in-depth interviews if invited.

We will not collect any biological samples from the study participants.

### Interventions

#### Explanation for the choice of comparators {6b}

**MILAP** is an innovative family-based intervention involving married woman, her husband, and MIL. Spanning nine weeks (11 total hours), MILAP addresses women’s current experiences of IPV and mental health needs by incorporating communication skills, stress management techniques, and strategies to strengthen relationships. We will compare MILAP with currently available IPV services, i.e., EUC delivered by WOREC. EUC builds on standard individual and group counseling by adding crisis counseling and streamlined referral mechanisms to ensure participants’ safety and facilitate access to comprehensive medical and psychosocial support.

As this is a 1:1 randomized trial, half of the total study participants (150 family triads; 450 individuals) will receive MILAP, while the remaining half will receive EUC.

#### Intervention description {11a}

##### Multi component family Intervention to Lower depression and Address Intimate Partner violence (MILAP):

The MILAP, summarized in Table 1 below, includes nine sessions delivered over nine weeks to young women aged 15–24, their husbands, and MILs.

All sessions will be delivered by counselors who have completed at least six months of psychosocial training and received specialized training to deliver MILAP from master’s-level psychologists and senior researchers. The sessions will take place in community-based counseling centers operated by either WOREC or local government, depending on participant convenience. MILAP is divided into three sections: MIL-DIL sessions, BCT sessions, and a triad summary session.

#### MIL-DIL sessions

MILAP begins with the MIL-DIL sessions for MIL-DIL dyad. The first two sessions (four total hours) are grounded in participatory learning and action principles using stories, role-plays, and discussions to enhance knowledge, skills, and social support. The primary focus of these two sessions will be to: a) discuss the impact of cultural gender norms, roles, and expectations; b) raise awareness about the negative impact of IPV on the family unit; c) establish allyship between MIL and DIL; and d) promote DIL’s empowerment and MIL’s support to improve the FOM of DILs.(72) These sessions also prepare participants for two one-hour sessions on brief behavioral activation (BA), an evidence-based technique for addressing and preventing depression.(73, 74) The BA sessionsfocus on enhancing women’s FOM and involve the MIL-DIL dyad in planning and engaging in rewarding activities,and providing positive reinforcement for DIL to experience positive feelings.

#### BCT sessions

After the completion of the MIL-DIL sessions, the husband-wife dyad will receive four weekly sessions of BCT (four total hours). The first two sessions focus on identifying shared challenges, committing to safety and trust, learning stress-coping strategies, and improving communication.(75) The third session introduces an activity to encourage caring behaviors between partners. The fourth session builds on communication skills by introducing active listening techniques and reviewing strategies learned in previous sessions.

#### Triad summary session

After completing the BCT sessions, the triad (woman, husband, and MIL) will participate in a one-hour summary session. This session will review key lessons from all previous sessions and prepare participants to address potential challenges in sustaining improvements in IPV and mental health.

Each session will incorporate role-plays, culturally grounded storytelling and reflections, regional and local folk songs, and will conclude with take-home assignments for participants to practice between sessions.

##### Enhanced usual care (EUC):

EUC will be delivered by a separate team of counselors who have completed at least six months of psychosocial training but have not received specialized MILAP training, in order to avoid potential contamination. These counselors will conduct an initial safety assessment and offer: a) WOREC’s IPV rehabilitation services, which include access to a women’s shelter, safety and protection from the perpetrator, mental health counseling, legal support, and health services; b) educational materials on safety planning, the consequences of ongoing IPV, written resources for reducing IPV, and information about a nationwide IPV/DV hotline; c) referrals to wraparound services based on the woman’s needs and priorities, including legal support, safe housing, psychosocial counseling, health services, and livelihood support; d) referral to a one-stop IPV center, along with information about available options, including support for leaving the relationship if the participant expresses that desire.

Since this study includes women currently experiencing IPV, we will enhance the usual care by adding two key services: mental health crisis counseling and a referral pathway for those in need of specialized support.

**Crisis counseling** will be provided to all participants, regardless of their intervention allocation, who are identified as being at high suicidality risk, as assessed by the study team members using the trigger (ninth) question of the PHQ-9 (“thoughts that you would be better off dead or thoughts of hurting yourself in some way”) and three questions from the NESS-3 that assess imminent suicide risk. Contracted crisis counselors will deliver these services, assess participants’ immediate risk, and provide urgent support as needed. If further care is required, study team members will facilitate referrals to tertiary care facilities. In cases where participants report escalated violence requiring immediate intervention, they will be referred to the One-Stop Crisis Management Center (OCMC) in their respective district. OCMC is a hospital-based center that provides a comprehensive range of services for survivors of gender-based violence, including medical services, psychosocial counseling, legal aid, personal security, rehabilitation and vocational training, coordination with safe homes, and connections to local community-based organizations working in the area of IPV.(76) If a woman doesn’t feel safe or doesn’t wish to return, study team members will connect her with WOREC for access to safe shelter or housing services.

#### Criteria for discontinuing or modifying allocated interventions {11b}

Participation in this trial will be voluntary and participants can discontinue at any time for any reason. Additionally, we will discontinue participants who are lost to follow-up due to various reasons, including relocation outside the study area or when the family triad no longer resides in the same household. Participants will be considered lost to follow-up if multiple contact attempts fail. These attempts include three consecutive phone calls (Days 1–3), two additional calls on alternate days (Days 5 and 7), attempts to contact to the woman’s listed emergency person (Days 8–10) and a confidential household visit to ensure participant’s well-being. If a missed assessment is found to be due to escalated violence or increased suicidality risk, we will follow established safety protocols and provide crisis management and referrals as needed.

We will not modify intervention allocation during the trial period. However, upon completion of the trial, we will offer and provide MILAP to interested EUC participants.

#### Strategies to improve adherence to interventions {11c}

We will employ several strategies to improve participants’ adherence to the intervention. First, we will provide detailed information about MILAP, including its objectives, content, target participants, and the potential benefits of each session, prior to enrollment. Second, we will collect comprehensive contact information from all participants at enrollment (and update it at follow-up assessments), including mobile phone numbers, email addresses (if available), street addresses, nearby landmarks, and the name and phone number of a reliable emergency contact (e.g. a friend or family member). This will help maximize our ability to contact participants for scheduling intervention sessions. Additionally, we will make two reminder calls, one 48 hours and another 24 hours before the scheduled session to minimize the likelihood of missed appointments. Third, we will prioritize participants’ comfort and availability when scheduling sessions and will provide modest compensation for their travel and time.

#### Relevant concomitant care permitted or prohibited during the trial {11d}

We will not prohibit participants from accessing any care during the trial.

#### Provisions for post-trial care {30}

We will offer MILAP to all EUC participants after the completion of trial, if they express interest in receiving the intervention. All participants, regardless of intervention allocation, will receive information on how to access other IPV and mental health services.

#### Outcomes {12}

This study has two primary outcomes. The first primary outcome is the change in the proportion of women with moderate to severe depression from baseline to the 12-month follow-up. To assess depression severity, we will use the PHQ-9, which has been validated in Nepal, and define moderate to severe depression as a PHQ-9 score of greater than nine.(70) The second primary outcome of interest is the change in IPV levels from baseline to the 12-month follow-up. We will assess IPV through measures of abusive control, physical violence, and sexual violence using the Indian Family Violence and Control Scale (IFVCS).(77) Our secondary outcome of interest is the severity of PTSD symptoms, measured by the PTSD CheckList-Civilian Version (PCL-C). Detailed information on primary, secondary and other outcome measures is presented in Table 2.

#### Participant timeline {13}

**Figure 2:**
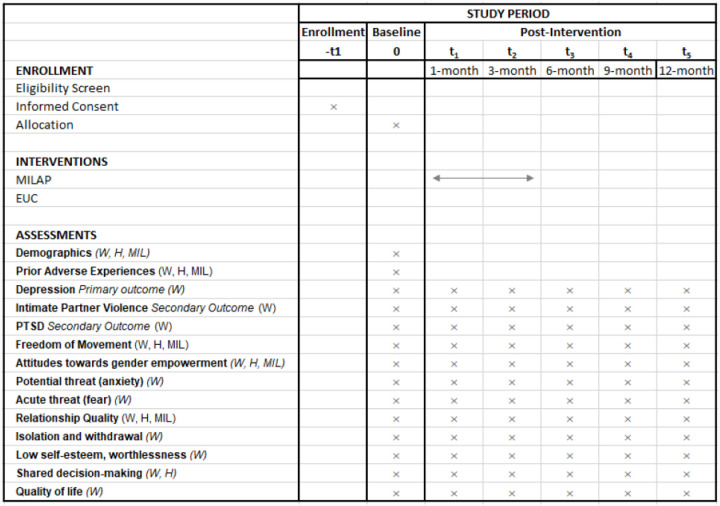
Study Timeline Note: W=Women, H=Husband, MIL=Mother-in-Law

After the consent process is completed, we will conduct baseline assessments. This will be followed by the 1:1 random allocation of the recruited participants to either MILAP or EUC. Participants will then receive the intervention based on their allocation. We will conduct follow-up assessments at 1, 3 6, 9, and 12 months from baseline for all participants, regardless of intervention allocation. To evaluate the effectiveness of individual MILAP components, the one-month follow-up assessment will take place immediately after the completion of all MIL-DIL sessions and prior to the start of the BCT sessions. The three-month follow-up will be conducted after participants have completed all nine MILAP sessions. Figure 2 below shows the study timeline.

#### Sample size {14}

Given the dual goals of the trial to assess MILAP’s effectiveness and understand mechanisms of action, sample size was determined for both mediation analyses and the impact on primary outcomes. Because mediation analysis is more statistically power-intensive, we calculated the sample size required to achieve 80% power to detect: a) the effect of the intervention on the primary outcome (the proportion of women with at least moderate depression), and b) a significant indirect effect of the intervention on depression, mediated by women’s FOM, one of the primary theorized mechanisms of change in our conceptual model. These calculations were based on the joint testing of both links in the hypothesized mechanistic pathway from intervention to FOM (link 1) and FOM to depression (link 2), using the approach of Vittinghoff and Neilands (78) and the corresponding medssp.R program. Based on our pilot study results, we expect the proportion of women with at least moderate depression (PHQ-9>9) post-intervention to be approximately 40% in the comparison group versus 5–10% in the intervention group. Based on the observed increase in FOM in the pilot study (65, 66), and to ensure clinical relevance, we specified a moderate effect of the intervention on the continuous FOM mediator, i.e., an R^2^ of 13% for FOM, per Cohen’s guidelines(79) for the first link, and an odds ratio of 0.5 per standard deviation (SD) of FOM for the dichotomous depression outcome for the second link of the indirect effect (i.e., a gain of 1 SD in FOM is associated with half the odds of scoring >9 on the PHQ-9). Assumptions for additional nuisance parameters that were necessary to be specified for this sample size calculation were: OR=0.77 for the direct effect of the intervention on depression (i.e., the remaining reduction in odds of PHQ-9 > 9 for the intervention group relative to EUC not mediated by FOM), and ρ_2_ = 0.30, a conventional moderate value (80) to account for correlation between the mediator and potential confounders. Given the 1:1 randomization, intervention exposure is 50% and it is reasonable to assume no confounding (ρ_1_ = 0) of the intervention-depression relationship. Based on these specifications, the required sample size was calculated to be n=251. To account for an anticipated attrition rate of approximately 15%, we determined an initial sample size of n=300 triads.

#### Recruitment {15}

To enroll a total of 300 families, we anticipate that research assistants will have to screen about 800 families. To facilitate recruitment, we will share study information with the Female Community Health Volunteers (trained local health volunteers who provide community-based education and services focused on maternal and child health, and family planning) (81, 82), groups from WOREC networks like mothers’ groups and adolescent groups, and local stakeholders like local municipal authorities. Additionally, we will establish a community advisory board comprising independent community representatives (e.g. local leaders, women human rights defenders, and representatives from local community-based organizations actively engaged in IPV and mental health-related work). The board will provide independent feedback and guidance to strengthen the study process, including recruitment strategies, and to help safeguard the safety of study participants.

**Figure 3:**
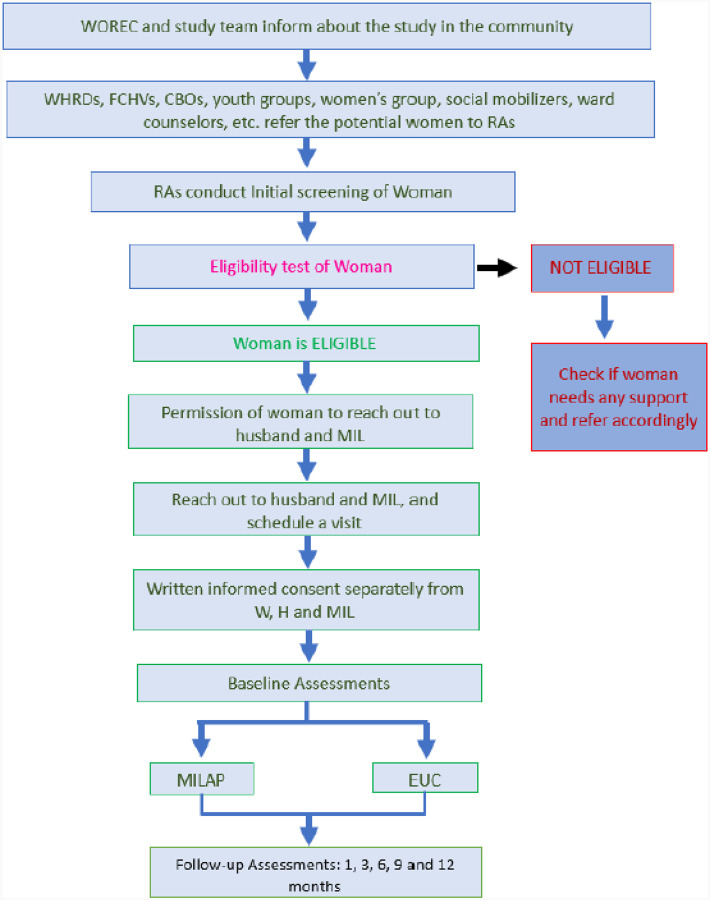
Recruitment Flow Note: WOREC: Women’s Rehabilitation Center WHRDs: Women Human Rights Defenders FCHVs: Female Community Health Volunteers CBOs: Community-Based Organizations RAs: Research Assistants W: Women H: Husband MIL: Mother-in-law EUC: Enhanced Usual Carev MILAP: Multi-component family intervention to lower depression and address intimate partner violence

After receiving a referral of potential woman participant, a research assistant will contact her and schedule an in-person appointment to assess eligibility. Given the stigma surrounding IPV and mental health, the research assistant will initially present the study as a family well-being intervention focused on reducing family conflict, and strengthening caring and supportive relationships. If the woman is eligible and provides permission to contact her family, the research assistant will then meet with her husband and mother-in-law (MIL) to confirm their eligibility. The recruitment flow is illustrated in Figure 3

### Assignment of interventions: allocation

#### Sequence generation {16a}

The data manager, who doesn’t have any direct interaction with participants and is not involved in data collection, will randomly assign each family triad to MILAP or EUC using a random number generator. We will conduct 1:1 randomization stratified by district.

#### Concealment mechanism {16b}

We will reveal intervention allocation to participants, research assistants, and counselors after randomization. We will, however, conceal the intervention allocation from assessment staff who conduct baseline and follow-up assessments. Assessment staff will be solely responsible for conducting baseline and follow-up assessments. They will not participate in any trainings, discussions, or activities that involve sharing information about the intervention. Research assistants will request the assessment staff to conduct the baseline and follow-up assessments as per the study timeline.

#### Implementation {16c}

Once the random allocation is generated, the data manager will reveal it first to the research coordinator who will then inform the research assistant using secured and confidential communication channel. The research assistant will then reveal the intervention allocation directly to participants via phone or in-person.

### Assignment of interventions: Blinding

#### Who will be blinded {17a}

The study team member who will conduct assessment, i.e. assessment staff, will be blinded. Since MILAP is a behavioral intervention that is unique (involving woman, husband, and MIL), participants receiving the intervention cannot be blinded. In addition, research assistants will not be blinded, as they are for responsible for coordinating with participants and counselors to schedule MILAP sessions.

#### Procedure for unblinding if needed {17b}

We do not anticipate needing to unblind.

### Data collection and management

#### Plans for assessment and collection of outcomes {18a}

We will use the REDCap platform to collect quantitative data. We will administer all assessment tools to both MILAP and EUC participants. We will administer demographic tool and prior adverse childhood experiences tool at baseline only. All other assessment tools will be administered at baseline and at follow-up time points: 1, 3, 6, 9, and 12 months post-baseline.

Depression will be measured using the widely used PHQ-9 tool, which is well-validated in Nepal.(70) We will use the IFVCS to measure abusive control, physical violence and sexual violence (77), and the PCL-C to measure PTSD.(83) Details of all outcomes measures, including demographic variables, are presented in Table 2.

To assess the mechanisms of change underlying MILAP’s effectiveness, we will collect qualitative data through serial in-depth interviews (IDIs) with all three members of the triad. We plan to conduct serial IDIs with approximately 15% of MILAP participants (n = 66), based on resource availability and the estimated number needed to reach thematic saturation (22 triads). We will conduct these serial IDIs at two timepoints: after the completion of MILAP and at the 12-month follow-up. We will purposively select triads to reflect variation in engagement with MILAP (e.g., across geographic areas and counselors), allowing us to capture a diverse range of intervention experiences. We will interview each participant separately using a semi-structured guide.

To conduct the cost-effectiveness analysis, we will collect data on the average costs per triad for delivering MILAP and EUC. We will also collect costs associated with each component of MILAP i.e. MIL-DIL, BCT, and the triad component. We will assess costs from a health system perspective, capturing both direct costs (e.g., staff time, training, materials, logistics) and indirect costs (e.g., monitoring, supervision, overhead, and administrative expenses) associated with implementation. In addition, we will capture costs incurred by participants and their households, including: (a) out-of-pocket expenses related to participation in the intervention (e.g., transportation and incidental costs), and (b) time costs, such as lost wages or productivity, due to time spent accessing and participating in the intervention.

#### Plans to promote participant retention and complete follow-up {18b}

We will utilize the strategies we have for intervention adherence (see above) for completing follow-up assessments. We will conduct assessments at a time and place that is convenient and preferred by participants. We will offer breaks as needed during assessments and provide a small financial incentive in appreciation of participants’ time.

#### Data management {19}

We will use the REDCap mobile app, which allows offline data collection using tablets. Trained research staff will collect and enter data on password-protected, encrypted tablets, and transfer it securely to the REDCap system, also password-protected, within 24 hours of data collection. We will conduct weekly data quality check to assess accuracy and completeness. Additionally, we will conduct monthly quality control checks to ensure data integrity and protocol adherence, identify any potential data issues early, and resolve them promptly. We will use STATA to process and manage quantitative data, and Dedoose for qualitative data management.

#### Confidentiality {27}

To ensure confidentiality in data collection and management, we will use the HIPAA-compliant REDCap mobile application on password-protected tablets for secure collection of responses. We have trained research assistants, assessment staff and counselors on maintaining participant confidentiality. We will assign different level of responsibility and REDCap login to safeguard data security, privacy and confidentiality. Login access will be managed through a two-factor authentication system and each user will be granted role-specific access (entry, view, or edit) based on their responsibilities within the study. To minimize potential contamination, we will implement several strategies: assign separate counselors to conduct MILAP and EUC sessions; restrict MILAP training to counselors delivering the intervention; and train both counselors and staff in controlled communication and contamination-mitigation techniques.Additionally, we have developed a research ethics brief to guide all study team members in the ethical conduct of the research involving vulnerable populations.(84)

#### Plans for collection, laboratory evaluation and storage of biological specimens for genetic or molecular analysis in this trial/future use {33}

Not applicable. We will not collect any biological specimens for genetic or molecular analysis in this trial/future use.

### Statistical methods

#### Statistical methods for primary and secondary outcomes {20a}

To evaluate the effectiveness of MILAP in reducing the proportion of women with moderate to severe depression and the levels of IPV and PTSD, we will conduct intention-to-treat (ITT) regression analyses at the end of the trial (12-month follow-up, single timepoint). Additionally, we will perform longitudinal analyses using a two-level regression model, incorporating an interaction between intervention arm and time, with repeated measures nested within participants (i.e., women) and a random intercept for participants. We will use logistic regression models for the binary outcomes (like depression defined by PHQ-9>9), and linear models for the continuous outcomes (like IFVCS score for IPV and PCL-C score for PTSD).

We will use thematic analysis to examine qualitative data from serial in-depth interviews (n = 66 across 22 triads). These interviews will help identify and prioritize potential mediators and moderators for quantitative analysis. We will triangulate the qualitative findings with quantitative data from the full cohort (n = 900 across 300 triads) and conduct a mixed-methods explanatory analysis to develop a comprehensive understanding of the mechanisms through which MILAP produces the intended outcomes and the relative contribution of each mechanism. To explore the mechanisms of change outlined in the conceptual model, we will conduct mediation analyses to assess whether, for example, the DIL-reported freedom of movement (FOM) score mediates the relation between intervention exposure and odds of DIL’s PHQ-9 >9. This will be examined by a structural equation model which simultaneously models the effect of the intervention on FOM (a), and of FOM (b) and intervention (c’) on depression, with a significant indirect effect (a*b) indicating mediation. (85) The model allows for continuous as well as categorical mediators and outcomes, and for multiple mediators to be included in the same model, to compare their relative impact.(86) We will use similar approach for other primary and secondary outcomes.

For cost-effectiveness analyses, we will conduct a trial-based cost-effectiveness analysis of MILAP at the 12-month follow-up, focusing on two primary outcomes: (1) cost per case of moderate to severe depression averted (PHQ-9 >9), and (2) cost per IPV event averted. Costs will be estimated from both health system and societal perspectives. We will use a bottom-up micro-costing approach at the family triad level to estimate intervention costs. At the facility level, we will apply time-driven activity-based costing, a participant-centered method that measures detailed resource use across service delivery processes.(87) We will also calculate incremental cost-effectiveness ratios by comparing differences in costs and outcomes between the MILAP and EUC groups.

#### Interim analyses {21b}

We will conduct interim analyses between follow-up assessments to monitor changes in IPV and depression severity to ensure that there is no unintended increase in either outcome. Additionally, we will document any adverse or serious adverse events to ensure participant safety and assess any potential risks associated with the MILAP.

#### Methods for additional analyses (e.g. subgroup analyses) {20b}

We do not plan to conduct any additional analyses other than those mentioned above.

#### Methods in analysis to handle protocol non-adherence and any statistical methods to handle missing data {20c}

The scenarios that are not covered by the trial protocol or those that require deviation from the protocol will be discussed within the team and will proceed with the approval of the principal investigators (PIs). We will document all such scenarios and decisions.

We will employ multiple imputation methods if missingness can be assumed to be (completely) at random for the primary analyses.(88) Alternatively, and depending on the type of analysis at hand (e.g. structural equation model), we will use full information maximum likelihood estimation.(89)

#### Plans to give access to the full protocol, participant level-data and statistical code {31c}

In compliance with the current National Institutes of Health Data Management and Sharing Policy, we will submit de-identified quantitative participant-level data to the National Institute of Mental Health (NIMH) Data Archive no later than one year after study completion. Additionally, we will share data and statistical code for quantitative analyses and codebook information for any qualitative data used in any publications at the time of publication.

### Oversight and monitoring

#### Composition of the coordinating centre and trial steering committee {5d}

Per the contractual obligations with the funder and institutional regulations, the tasks of coordinating centre and trial steering committee are conducted by a board described in the next section.

#### Composition of the data monitoring committee, its role and reporting structure {21a}

We will establish a five-member independent data safety and monitoring board led by an expert in mental health and IPV interventions. The board members will meet biannually to review safety procedures, ensure confidentiality protocols, and uphold participant protection standards.

### Safety monitoring & management

We will implement a standardized risk management system informed by findings from MILAP pilot studies, guidance from the community advisory board, and prior mental health research conducted by our team. During each intervention and assessment session, counselors and assessment staff will check in with participants regarding any recent incidents of IPV or other safety concerns. Any cases requiring urgent attention will be managed according to a pre-established crisis counseling and referral protocol.

During each assessment, assessment staff will administer PHQ-9, which includes a question on suicidality, to women participants. If a participant indicates any risk of suicidality, the assessment staff will administer the NESS-3. Patients identified as being at high and acute suicide risk will be immediately connected with a contracted crisis counselor for further evaluation and support. If immediate intervention is required, we will coordinate with the participant’s emergency contact and refer them to tertiary care facilities or OCMCs in their district. If no urgent referral is needed, the participant will be provided with a resource card containing contact information for relevant support services. In both scenarios, a study team member and the crisis counselor will follow up within 48 hours to ensure the participant’s safety and well-being. Severe IPV cases requiring medical attention will be referred to OCMCs at tertiary hospitals for mental health services, legal aid, medical care, shelters, and support from the community-based organizations. We will connect women in need of shelter support to WOREC for safe house services.

The study team will document all these incidents on standardized forms. The Principal Investigators (PIs) will review these reports and adjust safety protocols as needed. The data and safety monitoring board will conduct semi-annual evaluations to ensure the safety of participants throughout the study.

#### Adverse event reporting & harms {22}

The research coordinator will maintain an adverse event log and report incidents to research managers and the site PI. For serious adverse events, the research coordinator will immediately notify the site PI, who will inform the Ethical Review Board of the Nepal Health Research Council within 48 hours and submit a detailed report within two weeks. If multiple serious adverse events occur, the study team will review existing safety provisions and may temporarily pause the study. The site PI will also report serious adverse events to the Data and Safety Monitoring Board within three days and to the Institutional Review Board of the University of California San Francisco within 10 working days, in accordance with institutional guidelines. Adverse event determined to be unrelated to study participation will be securely stored at the study site office. The Data and Safety Monitoring Board will be responsible for determining whether an event is related to the study.

#### Frequency and plans for auditing trial conduct {23}

The site PI and research manager will conduct weekly reviews to monitor study implementation. The contact PI will perform monthly reviews to assess recruitment progress, participant retention, participant safety, and any methodological concerns. In accordance with reporting requirements, we will submit biannual recruitment and performance progress reports to the NIMH. Additionally, the study will undergo annual continuing ethical review by both the Nepal Health Research Council and the University of California San Francisco.

#### Plans for communicating important protocol amendments to relevant parties (e.g. trial participants, ethical committees) {25}

Any amendments to the trial protocol will be communicated to participants, members of the data safety and monitoring board, and relevant ethical/institutional review boards.

#### Dissemination plans {31a}

We will disseminate the findings of this study to multiple advisory boards, including the community advisory board and the data safety and monitoring board, as well as through various platforms such as peer-reviewed journals. We will share briefs, reports, and fact sheets to enhance accessibility. Additionally, we will present the study findings at regional, national, and international conferences to engage the broader research community. To support policy translation and impact, we will also conduct dissemination meetings with key stakeholders and policymakers following the completion of trial.

## Discussion

This study introduces MILAP, an innovative family-based intervention designed to address two deeply interconnected public health challenges, IPV and depression, which disproportionately burden young women in Nepal and other LMICs. In these settings, women often live in multi-generational households with limited autonomy and few options to leave abusive relationships. By leveraging family dynamics, MILAP offers a culturally grounded, non-stigmatizing approach that fosters resilience and empowerment at multiple levels. At the intrapersonal level, it equips women with essential coping and emotional regulation skills. At the interpersonal level, it strengthens bonds between MILs and DILs, and enhances spousal communication and trust. At the family level, it cultivates a supportive household environment that promotes women’s freedom of movement and overall well-being.

This randomized controlled trial will generate robust evidence on the effectiveness of family-based intervention like MILAP, that engages a woman, her husband and her MIL, to simultaneously reduce IPV and depression. In addition, we will conduct a cost-effectiveness analysis to estimate the health and economic benefits of scaling up MILAP in Nepal. The findings will inform decisions on broader implementation by demonstrating MILAP’s value relative to enhanced usual care in reducing IPV and improving mental health outcomes.

This study has multiple strengths. The intervention design, recruitment plans, retention strategies, and safety protocols are all informed by our pilot studies, input from the community advisory board and prior behavioral health research in the region. Rather than directly focusing on the stigmatized issues of IPV and depression, MILAP frames the intervention as a pathway to building healthier relationships, which was found to be acceptable and engaging in our pilot study. We have developed a robust recruitment and retention plan that leverages trusted community-based organizations, community advisory boards and mobilized community health workers to ensure sustained engagement. A known risk in IPV interventions is the potential escalation of violence. While pilot findings suggest MILAP does not increase IPV risk, we have developed rigorous safety protocol and will have a data safety and monitoring board set up to regularly review safety data.

The study may face some limitations. First, we will exclude severe IPV cases requiring hospitalization because such severe cases are unlikely to benefit from a behavioral intervention like MILAP. Although these cases represent a small minority, this exclusion limits the overall reach of the intervention. Second, although our pilot data showed high completion rates and the community advisory board confirmed that nine sessions are reasonable, there remains a risk that the completing all nine sessions may be burdensome for some participants. To reduce assessment-related burden, participants will receive modest compensation for their time and travel, and MILAP counselors will be provided with protected time to deliver MILAP.

IPV and mental health challenges require culturally responsive solutions that empower women while engaging key family influencers. MILAP has the potential to how IPV and depression are addressed, not only in Nepal but also in other socio-culturally similar contexts. If successful, this study will establish MILAP’s effectiveness, cost-effectiveness, and mechanism of change, shedding light on critical pathways such as FOM and women’s autonomy that drive positive transformations in IPV and mental health outcomes.

### Trial status

Protocol version 1.0, dated April 2, 2025. The recruitment period began on April 4, 2025, and we expect recruitment to be completed by March 2027.

## Figures and Tables

**Table 1:** 

Session Number	MILAP Session Type	Session Time	Focus	Participants
**1**	**MIL-DIL Sessions**	2 hours	Awareness of negative effects of IPV Discuss cultural norms and expectations Establish allyship	Woman and her MIL
**2**		2 hours	Gender empowerment Facilitate increased freedom of movement	
**3**		1 hour	Brief behavioral activation #1: list and plan rewarding activities	
**4**		1 hour	Brief behavioral activation #2: review experience + reinforce	
**5**	**Behavioral Couples Therapy (BCT) Sessions**	1 hour	Align on challenges, establish safety and trust Learn specific strategies to cope with stress	Couples: Woman and her Husband
**6**		1 hour	Revisit effectiveness of coping skills Understand and address communication challenges between partners	
**7**		1 hour	Activity to encourage caring behavior between partners Build communication skills	
**8**		1 hour	Active listening skills Review strategies to improve communication Addressing ongoing challenges, if necessary	
**9**	**Triad (Summary) Session**	1 hour	Review all lessons and prepare for long-term change	Woman, her husband and MIL

**Table 2:** List of Key Variables and Measurement Tools

Screening Tools	Baseline	Follow-up (I, 3, 6, 9, and 12 month)
1. Screening Tool (W) International Violence Against Women Sumy (IVAWS) Patient Health Questionnaire-9: first 2 questions and 9^th^ question. If the score of PHQ-9 first 2 questions >3, then administer all PHQ-9 I f PHQ-9 trigger Q 9 is “More than half the days” or “Nearly every days”, then Administer 3 questions of Nepal Emergency Suicide Screener (NESS) Severity of Alcohol Dependence Questionnaire (SADQ)	1. Demographic Questions (W, H, MIL)	
	2. SRQS 21-item: Spousal relationship quality score (21 items) (W, H, MIL)	1. SQRS 21-item: Spousal quality relationship scale (21 items) (W, H, MIL)
	3. MILRQS 7-item: Mother-in-Law Relationship Quality Score (7 items) (W, H, MIL)	2. MILRQS 7-item: Mother-inLaw Relationship Quality Scale (7 items) (W, H, MIL)
	4. WAS 5-item: Women’s Autonomy Scale (4 items) (W, H, MIL)	3. WAS 5-item: Women’s Autonomy Scale measuring Freedom of Movement (4 items) (W, H, MIL)
	5. WHOQOL-BREF 9-item (psychological well-being and social functioning) (W)	4. WHOQOL-BREF 9-item (psychological well-being and social functioning) (W)
2. Severity of Alcohol Dependence Questionnaire (SADQ) for Husband and MIL (H, MIL)	6. RSES 10-item: Rosenberg Self-Esteem Scale (10 items) (W)	5. RSES 10-item: Rosenberg Self-Esteem Scale (10 items) (W)
	7. FSS Fear Survey Schedule (focused on injury and sexual aggression fear) (W)	6. FSS: Fear Survey Schedule (focused on injury and sexual aggression fear) (W)
	8. ASI 16-rttm (Anxiety Sensitivity Index) (W)	7. ASI 16-item (Anxiety Sensitivity Index) (W)
	9. GAD-7: Generalized Anxiety Disorder-7 (W)	8. GAD-7: General ized Anxiety Disorder-7 (W)
	10. PHQ-9: Patient Health Questionnaire-9 & NEpal Suicide Screener (NESS) (W)	9. PHQ-9: Patient Health Questionnaire-9 & NESS (W)
	11. PCL-C 17-item: PTSD Checklist for DSM-4 (W)	10. PCL-C 17-item: PTSD Checklist for DSM-4 (W)
	12. BAD5–5F 9-ilem: Behavioral Actuation for Depression Scale - (9 items) (W)	11. BADS-SF 9-item: Behavioral Activation for Depression Scale - (9 items) (W)
	13. IFVCS 63-item: Indian Family Violence and Control Scale (63 items) (W)	12. IFVCS 63-item: Indian Family Violence and Control Scale (G3 items) (W)
	14. SRPS 8-item: Sexual relationship power scale (8 items) (W, H)	13. SRPS S-item. Sexual relationship power scale (8 items) (W, H)
	15. Adverse Childhood Experiences (W, H, MIL)	
	16. Additional 3 question from IVAWS on experiencing IPV to Mother-in-law (MIL)	
	17. GEMS 4-item: Gender Equality Men Scale (4 items) (W, H, MIL)	14. GEMS 4-item: Gender Equality Men Scale (4 items) (W, H, MIL)
	18. WHODAS 2.0 12-item: World Health Organization Disability Assessment Schedule 2.0 (12 items) (W)	15. WHOOAS 2.0 12-item: World Health Organization Disability Assessment Schedule 2.0 (12 items) (W)

## Data Availability

The de-identified participant-level data will be shared with the National Institute of Mental Health (NIMH) Data Archive. We will publicly share all statistical codes when sharing findings of this study.

## Abbreviations

AE: Adverse Event
BCT: Behavioral Couples Therapy
CBT: Cognitive Behavioral Therapy
DIL: Daughter-in-Law
DM: Data Manager
EUC: Enhanced Usual Care
FOM: Freedom of Movement
HIPAA: Health Insurance Portability and Accountability Act
IDI: In-depth Interview
IFVCS: Indian Family Violence and Control Scale
IPV: Intimate Partner Violence
ITT: Intention-to-Treat
IVAWS: International Violence Against Women Survey
LMIC: Low and Middle-Income Country
MIL: Mother-in-Law
MILAP: **M**ulti component family **I**ntervention to **L**ower depression and **A**ddress intimate **P**artner violence
NESS: Nepal Emergency Suicide Screener
NIMH: National Institute of Mental Health
OCMC: One stop Crisis Management Center
PCL-C: Post-traumatic Stress Disorder (PTSD) CheckList-Civilian Version
PHQ-9: Patient Health Questionnaire-9
PI: Principal Investigator
PTSD: Post-traumatic Stress Disorder
RCT: Randomized Controlled Trial
REDCap: Research Electronic Data Capture
SADQ: Severity of Alcohol Dependence Questionnaire
SCT: Social Cognitive Theory
SD: Standard Deviation
WOREC: Women Rehabilitation Center
